# WHAT MAKES HUMAN HEARING SPECIAL?

**DOI:** 10.3389/frym.2022.708921

**Published:** 2022-05-31

**Authors:** Christian J. Sumner, Christopher Bergevin, Andrew J. Oxenham, Christopher A. Shera

**Affiliations:** 1 NTU Psychology, Nottingham Trent University, Nottingham, United Kingdom; 2 Department of Physics and Astronomy and Centre for Vision Research, York University, Toronto, ON, Canada; 3 Department of Psychology, University of Minnesota, Minneapolis, MN, United States; 4 Department of Otolaryngology, University of Minnesota, Minneapolis, MN, United States; 5 Caruso Department of Otolaryngology, University of Southern California, Los Angeles, CA, United States; 6 Department of Physics and Astronomy, University of Southern California, Los Angeles, CA, United States

## Abstract

Humans and many other animals can hear a wide range of sounds. We can hear low and high notes and both quiet and loud sounds. We are also very good at telling the difference between sounds that are similar, like the speech sounds “argh” and “ah,” and picking apart sounds that are mixed together, like when an orchestra is playing. But how do human hearing abilities compare to those of other animals? In this article, we discover how the inner ear determines hearing abilities. Many other mammals can hear very high notes that we cannot, and some can hear quiet sounds that we cannot. However, humans may be better than any other species at distinguishing similar sounds. We know this because, milliseconds after the sounds around us go into our ears, other sounds come *out*: sounds that are actually produced by those same ears!

## INTRODUCTION

Our ears allow us to communicate with one another and to explore the world around us. They enable us to understand speech, hear music, and keep ourselves safe. They help other animals hunt, eat, and avoid being eaten. Most of the work of hearing is done inside our heads, in the inner ear, which contains the most intricate and rapidly moving parts in the body. Sound moves through the air as vibrations and is caught by the part of the ear we can see—the outer ear. The vibrations then move down the ear canal, through the middle ear, and into the inner ear. Here, the vibrations are converted into electrical signals that travel along nerves to the brain. The brain then works out what is making the sound, where the sound is coming from, and whether we need to do anything about it. Hearing these vibrations tells us that something out there is making a sound, but it also tells us a lot about the sound: is it an adult asking us if we have done our homework, or a friend asking whether we want a cookie? Most animals can hear some sounds that humans cannot hear, but it turns out that human hearing is remarkable in a different way.

## SOUNDS PLAY THE “PIANO KEYBOARD” IN THE EAR

**Figure 1:**
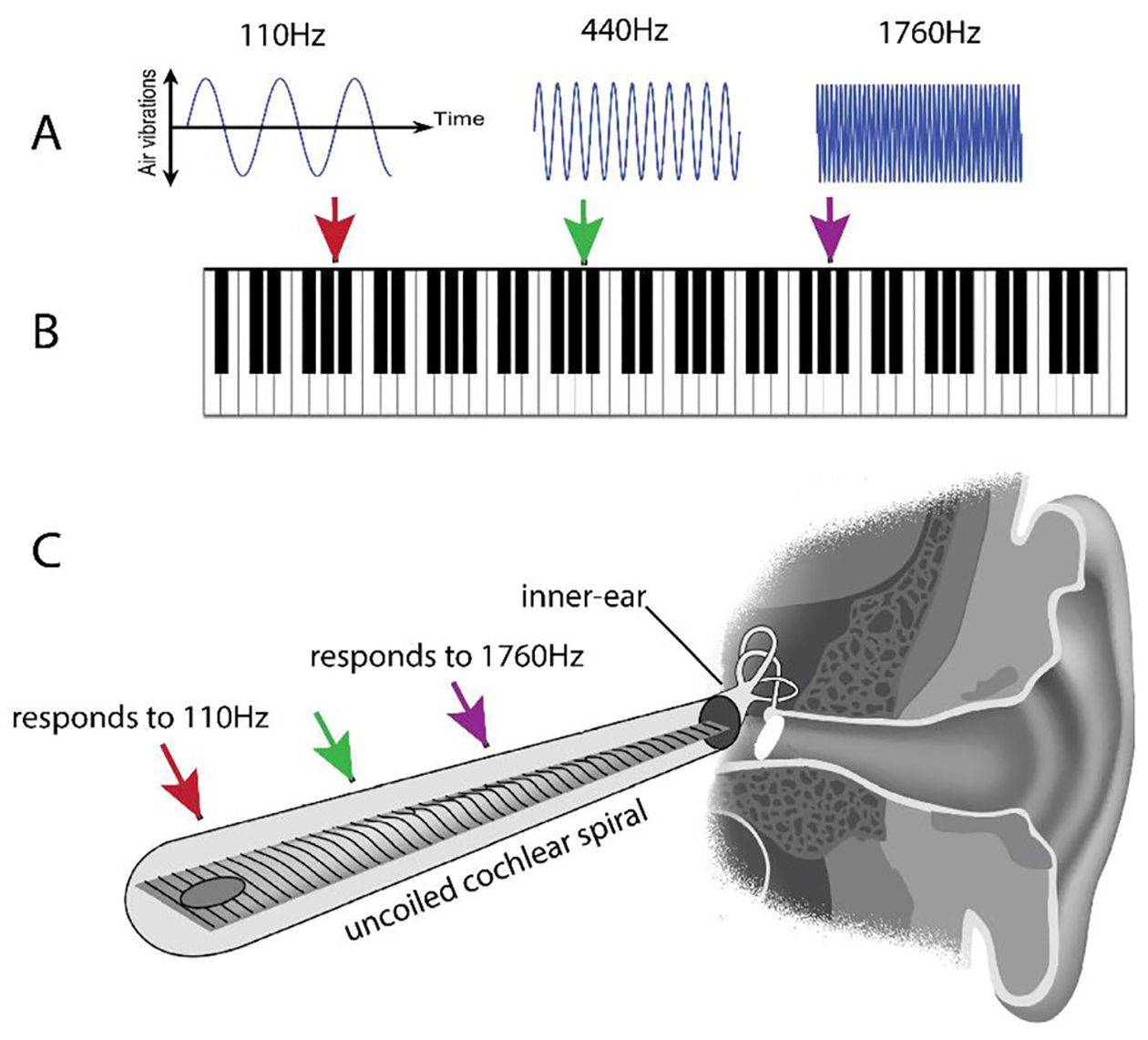
**(A)** Low-frequency sounds (left) make the air vibrate slower and high-frequency sounds (right) make the air vibrate faster. **(B)** These sound frequencies match with the musical notes on a piano keyboard. (C) Specific sound frequencies map to different locations in the inner ear (cochlea), shown in the cut-away of a human head. The cochlea is a spiral structure, like a snail shell, and specific frequencies cause nerve fibers to fire at specific points along the spiral. Here the cochlea is shown “uncoiled,” to show the mapping of sound frequencies [image credit: **(C)** modified from a Wikipedia Creative Commons license image].

The sounds that travel as vibrations to the inner ear vary a lot—that is why they sound different! One important way that vibrations vary is in how fast the air is vibrating (Figure 1A). Imagine a piano keyboard (Figure 1B). The notes on the left side of the keyboard cause the strings inside the piano (and therefore the air around them) to vibrate slowly. To us, those notes sound “low.” The notes on the right of the keyboard produce fast vibrations and sound “high” to us. We call the speed of these vibrations the **frequency** of the sound. We measure frequency in Hertz (abbreviated Hz), which is the number of vibrations per second. We are remarkably sensitive to the frequency of vibration, and we perceive different frequencies as different musical notes, or “pitches.” How do we do that?

**FREQUENCY**

The rate of vibration of sound waves in the air. The number of times per second that air molecules complete a cycle of being squashed together, expand out, and back.

Incredibly, our ears sort out sounds from low to high, much like a piano keyboard! This important discovery was made almost 100 years ago by the Hungarian scientist Georg von Bekesy. It won him a Nobel Prize in 1961. The inner ear, or **cochlea**, is a spiral tube, shaped like a snail shell. Along the spiral is a **sound frequency map**, laid out like a piano keyboard. Figure 1C shows what the cochlear spiral would look like if it were pulled straight.

**COCHLEA**

The spiral shaped structure of the inner ear, where sounds are detected and organized according to frequency.

**SOUND FREQUENCY MAP**

**Figure 2:**
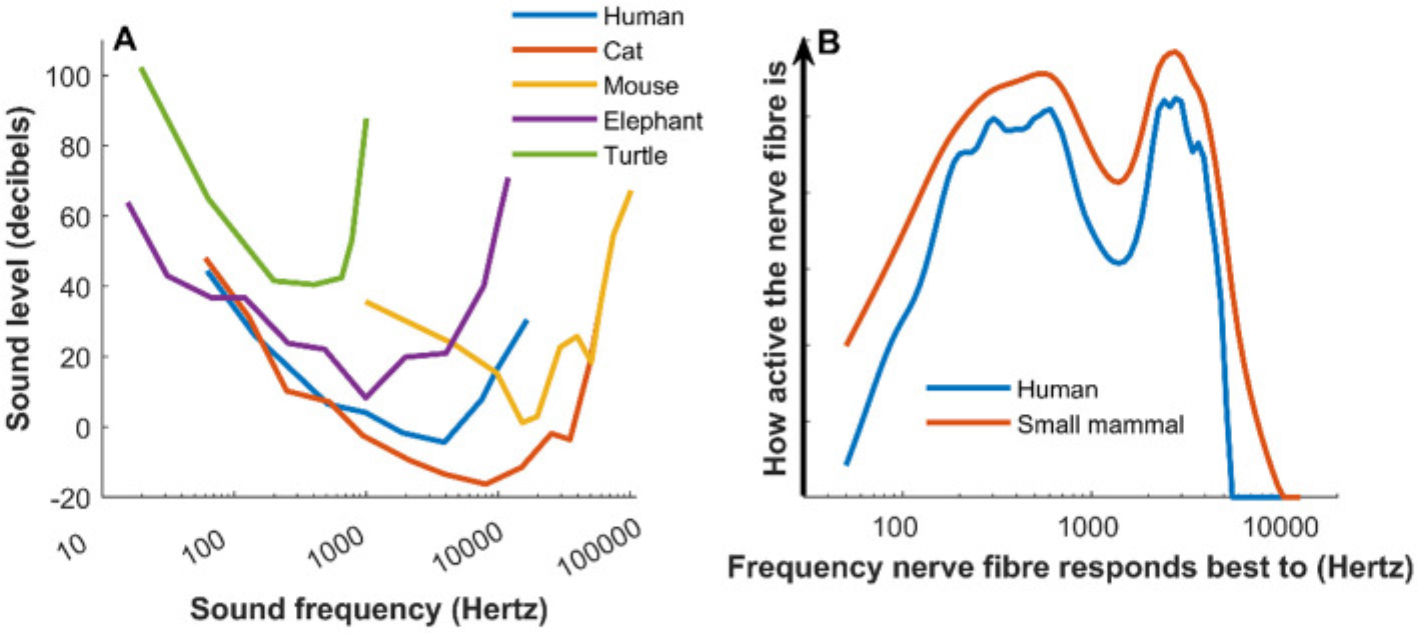
**(A)** Audiograms of various species show that humans fall squarely in the middle in terms of frequency range and sensitivity of hearing. Cats and mice, are more sensitive to higher frequencies. Cats are more sensitive to quiet sounds than humans are, while, elephants and turtles, are less sensitive overall (figure credit: [1]). **(B)** A computer simulation of activity in auditory nerve fibers in a small mammal and human, in response to the vowel sound “ee” as in “need.” This graph shows that activity in human nerve fibers gives more detailed information about sounds than those of other mammals.

Responses to sound are physically organized along the length of the cochlea, or across the surface of the brain, with orderly increasing sound frequency, like a graph (see Figure 2B).

Of course, the job of the cochlea is to *detect sounds*, not to make sounds like a piano. Instead of piano strings that *make* sounds of varying frequencies, the cochlea has locations along its spiral that *detect* vibrations of specific frequencies. Each location is most sensitive to a particular frequency and is connected to specific nerve fibers that send signals to the brain. The brain then works out the frequencies of the sounds we hear by determining which set of nerves is sending the signal.

The cochlea sends a constant chatter of electrical signals to the brain, telling us about the frequencies of sounds in the air as they change over time. The sound from an orchestra, or from human speech, is a complex combination of vibrations—lots of frequencies all at once! Somehow, we make sense of these complex sounds most of the time.

## YOUR PETS HEAR SOUNDS THAT YOU CANNOT!

Comparing ourselves to other animals can help us better understand how hearing works. There are a few ways to measure how “good” our hearing is. One way is to look at the range of low to high sounds we can hear. Although humans cannot hear all frequencies, we can hear sounds both higher and lower in frequency than the notes on the piano. In fact, a keyboard covering the entire frequency range that a young human ear can hear (from 20 to 20,000 Hz, or roughly 10 octaves) would require about 120 keys instead of the 88 found on a grand piano (32 more—you would need long arms!). However, some animals can hear much higher frequencies than people can. Cats and dogs can hear frequencies twice as high as humans (about 40,000 Hz). Mice hear in the ultrasonic range (up to about 80,000 Hz) but cannot really hear frequencies below 1,000 Hz, which are the frequencies important for human speech and music (look back to Figure 1 to see how much of the piano keyboard that is).

We can also look at how sensitive our hearing is. This is a measure of how well we can hear very quiet sounds. People and other animals are best at hearing quiet sounds in the middle of their range, not at the upper or lower ends. We can use a graph called an **audiogram** to illustrate, for each frequency, how intense a sound must be to be heard (Figure 2A). The intensity of a sound is measured in decibels, where zero is approximately the quietest sound we can hear, and 100 can be uncomfortably loud. The audiograms show big differences in the frequencies that various animals can hear, but they also show that most animals are similarly sensitive to the quietest sounds. So, in terms of their sensitivity to quiet sounds and the frequency range of their hearing, humans are very ordinary: other animals can hear higher frequencies than we can, lower frequencies than we can, or quieter sounds than we can.

**AUDIOGRAM**

A graph showing for each frequency sound, how intense it must be to be heard at all. Sounds below this level are undetectable.

As you might expect, hearing is linked to an animal’s size, its environment, and its communication needs. Human hearing is most sensitive to the frequencies present in human speech. Mice are small and produce high-pitched, squeaky sounds that we (thankfully) cannot hear. Not only can we not hear what mice are saying, but mice cannot hear much of what we are saying, either.

## YOU MIGHT BE BETTER THAN YOUR PET AT TELLING SOUNDS APART

Being able to detect sounds tells us that *something* is out there, but we need to know more. Is it a bus that might run you over, or a friend asking you to play? Imagine your friend is talking with the television on in the background. Can you separate the words coming from the TV from the words your friend is speaking? How selective your hearing is to sounds of similar frequencies affects how easy or di cult it is to tell sounds apart. This **frequency selectivity** also affects how well you can distinguish between different sounds, like the words “go” and “slow.”

**FREQUENCY SELECTIVITY**

Different parts of the cochlea respond to different frequencies. In a more selective cochlea, each part of the cochlea responds to a smaller range of frequencies.

Until recently, scientists thought that all mammals had similar frequency selectivity. It now seems that human hearing has better frequency selectivity than the hearing of most other species. Humans quite possibly hold the world record in this respect [2]! Figure 2B shows computer simulations of how scientists think the pattern of nerve activity varies between humans and small mammals when listening to the vowel sound “ee” (as in the second syllable of “cookie”). The human ear is selective enough to reveal important details in the sound, which shows up as small variations in the activity of nerve fibers. The more frequency selective an animal’s hearing is, the more detailed the pattern of nerve activity when it hears the sound.

## COMPARING SELECTIVITY IN DIFFERENT SPECIES

When testing hearing, it is difficult to measure humans and animals the same way. In humans, we can easily ask, “Is sound A different from sound B?” By varying the frequency of sounds, we can measure how accurately humans can distinguish various frequencies. It is not so easy to ask a mouse or a dog such questions! On the other hand, in animals we can directly monitor how the nerve fibers and brain cells respond to different sounds. This usually involves complex brain surgery, which makes it unsuitable for use in people.

Because the measurement methods for selectivity are different for humans and animals, it can be challenging to compare results across species. When we see differences, we must ask whether they are *real* differences in hearing or just a result of using different measurement techniques. It is possible to train animals to perform hearing tests similar to those used on humans, but it is very difficult and timing consuming. Your dog might sit down on command but imagine the difficulty of training it to sit or give you a paw when it hears two adjacent notes on the piano! Recording from the nerves of the inner ear may one day be possible in humans, but it is very difficult and has not yet been achieved.

A solution to this dilemma came from a rather surprising technique. In 1978, a scientist named David Kemp discovered that sounds do not just go *into* the ear, they come *out* as well! When a sound enters the inner ear, the sensory cells of the cochlea pick up the vibrations and then add more vibrations of their own, which bounce back out of the ear like an echo. These are called **otoacoustic emissions** and they are often used to test the hearing of newborn babies. Otoacoustic emissions can also be used to probe the frequency selectivity of the cochlea, although it involves some rather complicated mathematics. In a nutshell, the longer it takes a sound to come back out of the ear, the more frequency selective the cochlea is, and therefore the more different are the patterns of nerve firings in response to different frequencies.

**OTOACOUSTIC EMISSIONS**

Sounds that are produced by the inner ear vibrating. These can be spontaneous or produced in response to sound. Vibrations in the inner ear result in sound at the outer ear.

**Figure 3:**
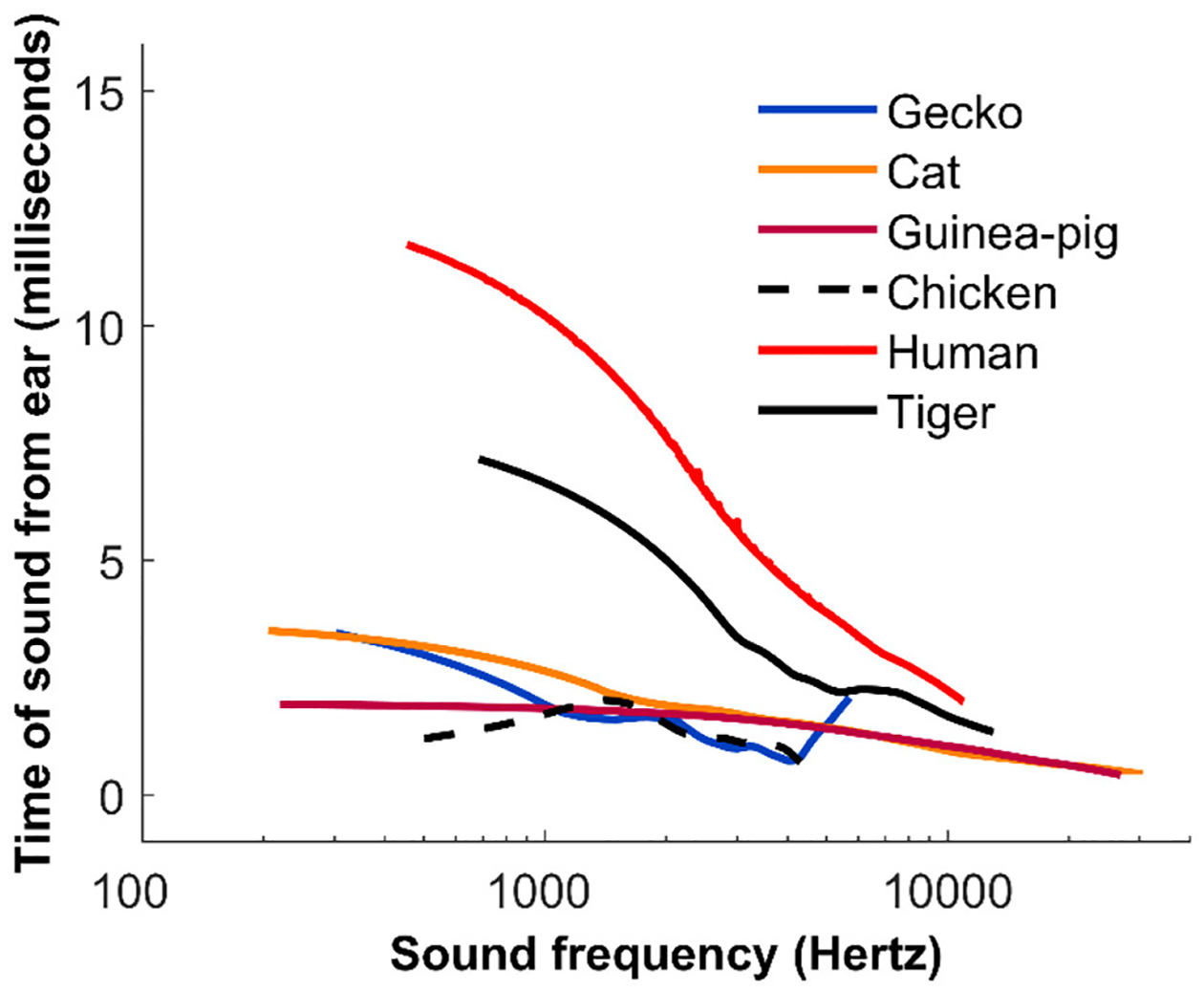
Measurements from the sounds (otoacoustic emissions) coming *out* of the ears of various species. The y-axis shows how long it takes after a sound is played into the ear before the response comes out. The longer it takes, the more selectively each nerve fiber responds to a particular range of frequencies (figure credit: [3]).

With this in mind, some of our group played tones of various frequencies to the ears of different animals and recorded the otoacoustic emissions (Figure 3) [3]. The measurements suggested that human ears are *more* frequency selective than the ears of other animals. More recently, we carefully explored whether measuring otoacoustic emissions is accurate, by painstakingly making all the different types of measurements we have talked about here (perception, otoacoustic emissions, and nerve recordings) in a single species: the ferret [4]. Ferrets are relatively easy to train and have a hearing range similar to that of humans. These measurements confirmed that otoacoustic emissions *do* show how selective the inner ear is to sounds of various frequencies. Most scientists now agree that, while human ears may not hear the high frequencies audible to some of our mammalian relatives, we have better frequency selectivity than most of those animals.

## WHY SHARP HEARING IN HUMANS?

Although many species can hear sounds that we cannot, it certainly looks like humans are better at separating sounds and telling similar sounds apart than other species are. Perhaps this ability is related to our amazing skills in communicating. Outstanding frequency selectivity may well have played a role in human evolution and the development of language and communication [see this Frontiers for Young Minds article [5], and this one [6], for examples of the subtle ways we use and recognize speech]. However, nothing is ever totally simple. Surprisingly, humans can still understand speech even when the sounds have been computer modified to remove most of the frequency differences [7]. Also, a few species, such as tigers, do not speak like we do but appear to have hearing that is almost as selective as that of humans (Figure 3). The next challenge is to figure out *why* human hearing is so selective.

To sum up, we have known for a long time that in terms of range of frequencies and sensitivity to quiet sounds, a human would not win the top prize in a “spot-the-sound” competition against other animals. There would likely be another animal that could spot a quiet sound more easily than us! However, we now know that human hearing is more frequency selective than other animals. So perhaps we could win the inter-species “spot-the-difference-between-sounds” competition!

## References

[1] Dent M 2017. Animal psychoacoustics. Acoust. Today 13:19–26. Available online at: https://acousticstoday.org/wp-content/uploads/2017/08/Dent.pdf

[2] Shera CA, Guinan JJ Jr., and Oxenham AJ 2002. Revised estimates of human cochlear tuning from otoacoustic and behavioral measurements. Proc. Natl. Acad. Sci. U. S. A 99:3318–23. doi: 10.1073/pnas.032675099 11867706 PMC122516

[3] Bergevin C, Verhulst S, and van Dijk P 2017. Remote sensing the cochlea: otoacoustics. Understanding the Cochlea 62:287–318. doi: 10.1007/978-3-319-52073-5_10

[4] Sumner CJ, Wells T, Bergevin C, Sollini J, Palmer AR, Oxehham AJ, 2018. Convergent measures of mammalian cochlear tuning confirm sharper human tuning. Proc. Natl. Acad. Sci. U. S. A 115:11322–6. doi: 10.1073/pnas.1810766115 30322908 PMC6217411

[5] Everhardt M, Sarampalis A, Coler M, Başkent D, and Lowie W 2022. Speech prosody: the musical, magical quality of speech. Front. Young Minds 10:698575. doi: 10.3389/frym.2021.698575

[6] Smith H, Pautz N, and Mueller-Johnson K 2021. Is it possible to identify a criminal by voice alone? Front. Young Minds 9:689812. doi: 10.3389/frym.2021.689812

[7] Shannon R, Zeng F, Kamath V, Wygonski J, and Ekelid M 1995. Speech recognition with primarily temporal cues. Science 270:303–4. 7569981 10.1126/science.270.5234.303

